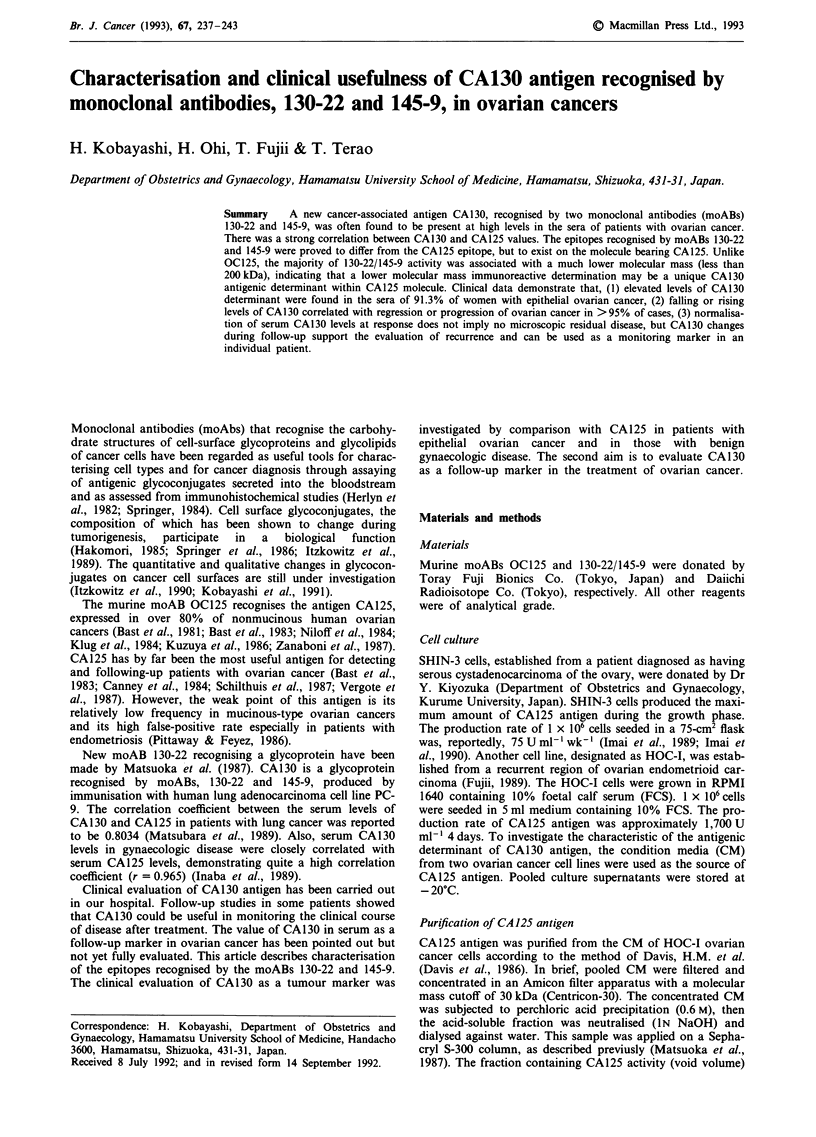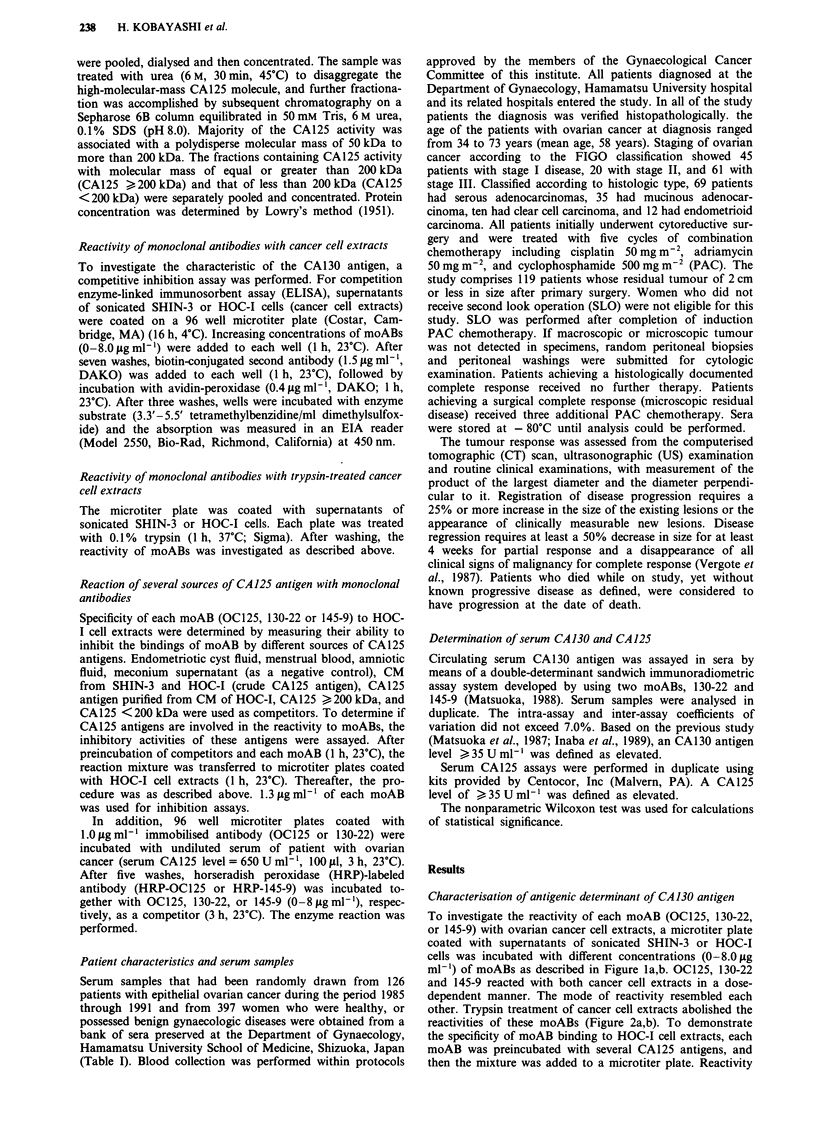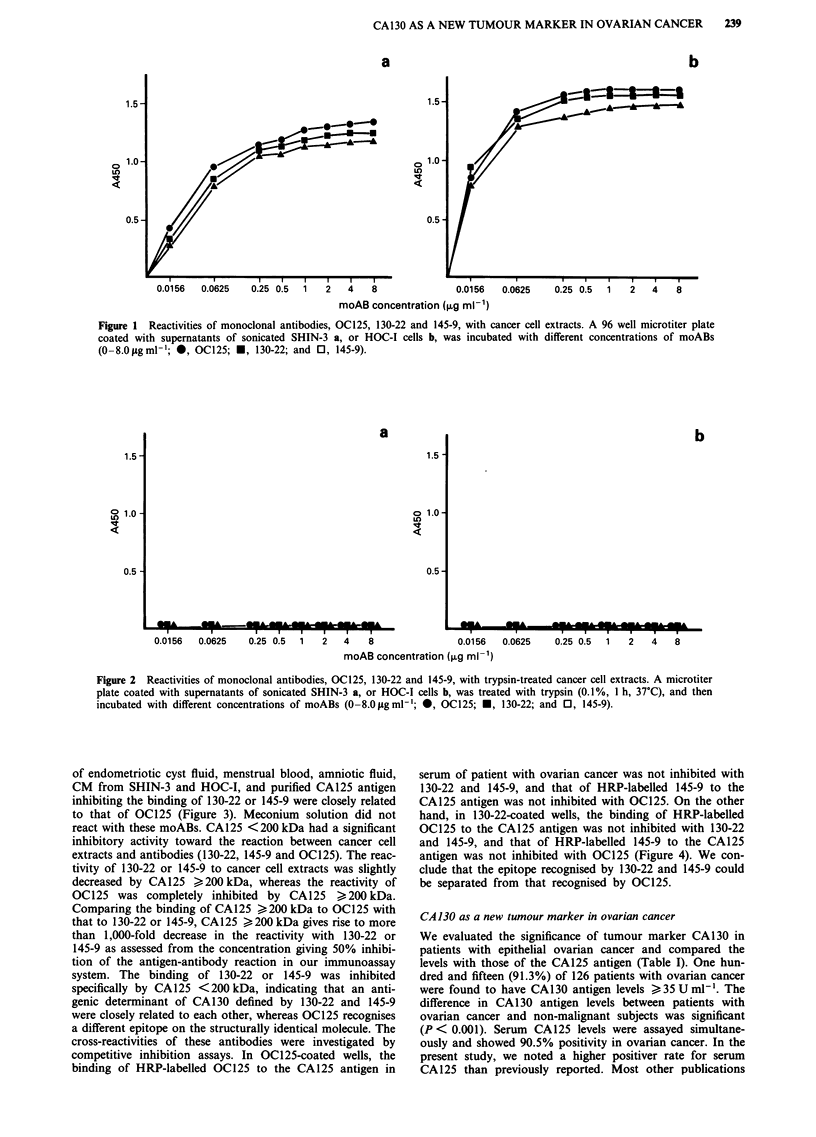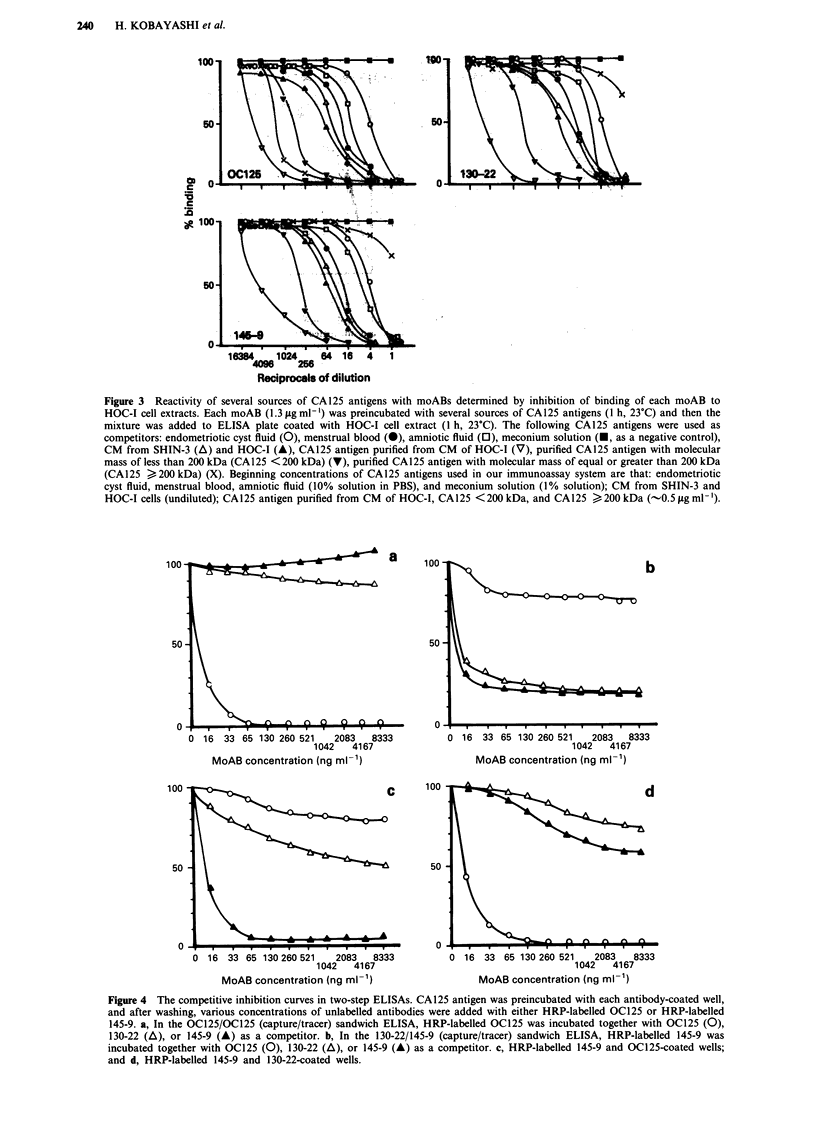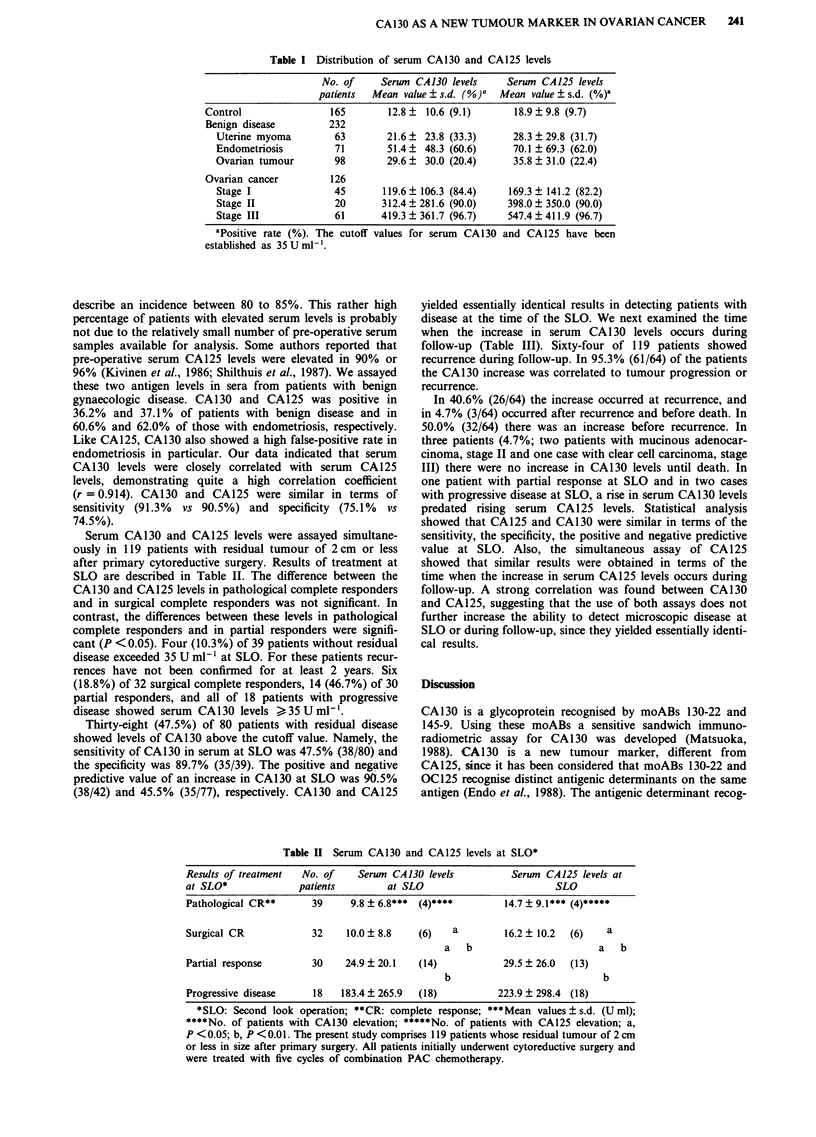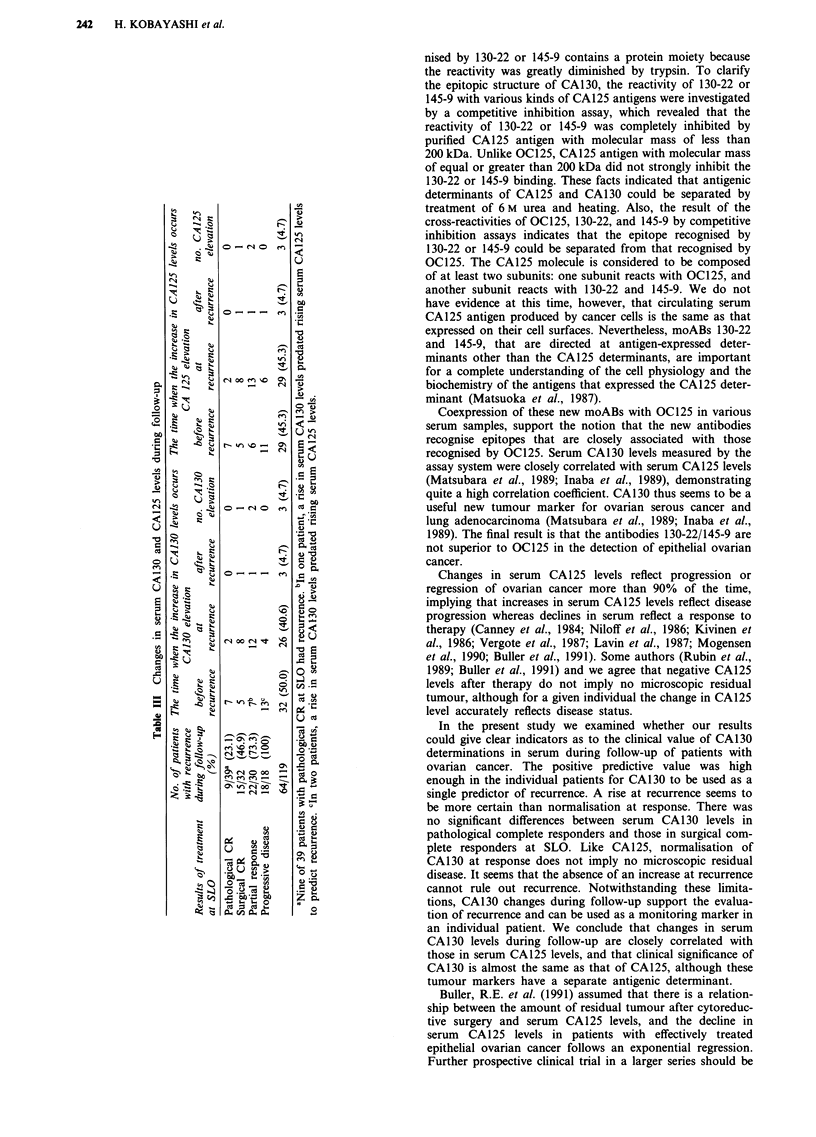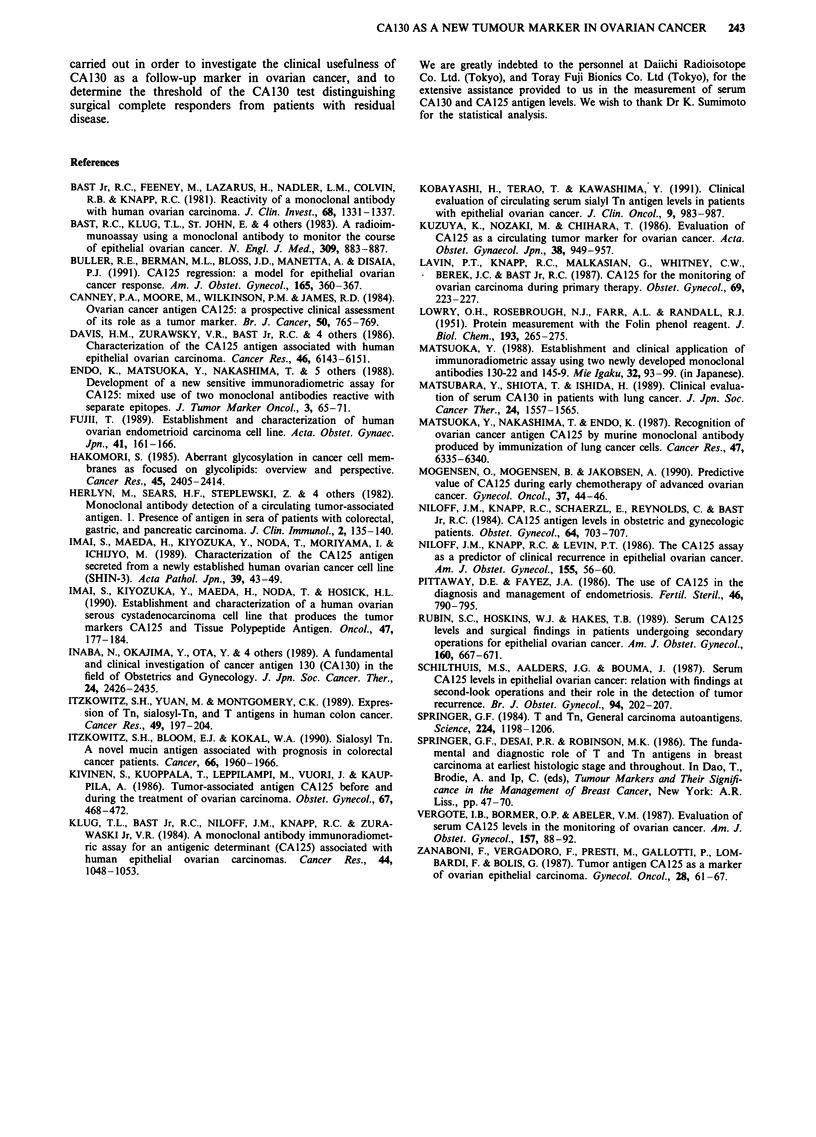# Characterisation and clinical usefulness of CA130 antigen recognised by monoclonal antibodies, 130-22 and 145-9, in ovarian cancers.

**DOI:** 10.1038/bjc.1993.46

**Published:** 1993-02

**Authors:** H. Kobayashi, H. Ohi, T. Fujii, T. Terao

**Affiliations:** Department of Obstetrics and Gynaecology, Hamamatsu University School of Medicine, Shizuoka, Japan.

## Abstract

A new cancer-associated antigen CA130, recognised by two monoclonal antibodies (moABs) 130-22 and 145-9, was often found to be present at high levels in the sera of patients with ovarian cancer. There was a strong correlation between CA130 and CA125 values. The epitopes recognised by moABs 130-22 and 145-9 were proved to differ from the CA125 epitope, but to exist on the molecule bearing CA125. Unlike OC125, the majority of 130-22/145-9 activity was associated with a much lower molecular mass (less than 200 kDa), indicating that a lower molecular mass immunoreactive determination may be a unique CA130 antigenic determinant within CA125 molecule. Clinical data demonstrate that, (1) elevated levels of CA130 determinant were found in the sera of 91.3% of women with epithelial ovarian cancer, (2) falling or rising levels of CA130 correlated with regression or progression of ovarian cancer in > 95% of cases, (3) normalisation of serum CA130 levels at response does not imply no microscopic residual disease, but CA130 changes during follow-up support the evaluation of recurrence and can be used as a monitoring marker in an individual patient.


*Br. J. Cancer* (1993), **67**, 237–243 © Macmillan Press Ltd., 1993

### Characterisation and clinical usefulness of CA130 antigen recognised by monoclonal antibodies, 130-22 and 145-9, in ovarian cancers

H. Kobayashi, H. Ohi, T. Fujii & T. Terao

*Department of Obstetrics and Gynaecology, Hamamatsu University School of Medicine, Hamamatsu, Shizuoka, 431-31, Japan.*

**Summary** A new cancer-associated antigen CA130, recognised by two monoclonal antibodies (moABs) 130-22 and 145-9, was often found to be present at high levels in the sera of patients with ovarian cancer. There was a strong correlation between CA130 and CA125 values. The epitopes recognised by moABs 130-22 and 145-9 were proved to differ from the CA125 epitope, but to exist on the molecule bearing CA125. Unlike OC125, the majority of 130-22/145-9 activity was associated with a much lower molecular mass (less than 200 kDa), indicating that a lower molecular mass immunoreactive determination may be a unique CA130 antigenic determinant within CA125 molecule. Clinical data demonstrate that, (1) elevated levels of CA130 determinant were found in the sera of 91.3% of women with epithelial ovarian cancer, (2) falling or rising levels of CA130 correlated with regression or progression of ovarian cancer in >95% of cases, (3) normalisation of serum CA130 levels at response does not imply no microscopic residual disease, but CA130 changes during follow-up support the evaluation of recurrence and can be used as a monitoring marker in an individual patient.

Monoclonal antibodies (moAbs) that recognise the carbohydrate structures of cell-surface glycoproteins and glycolipids of cancer cells have been regarded as useful tools for characterising cell types and for cancer diagnosis through assaying of antigenic glycoconjugates secreted into the bloodstream and as assessed from immunohistochemical studies (Herlyn *et al.*, 1982; Springer, 1984). Cell surface glycoconjugates, the composition of which has been shown to change during tumorigenesis, participate in a biological function (Hakomori, 1985; Springer *et al.*, 1986; Itzkowitz *et al.*, 1989). The quantitative and qualitative changes in glycoconjugates on cancer cell surfaces are still under investigation (Itzkowitz *et al.*, 1990; Kobayashi *et al.*, 1991).

The murine moAB OC125 recognises the antigen CA125, expressed in over 80% of nonmucinous human ovarian cancers (Bast *et al.*, 1981; Bast *et al.*, 1983; Niloff *et al.*, 1984; Klug *et al.*, 1984; Kuzuya *et al.*, 1986; Zanaboni *et al.*, 1987). CA125 has by far been the most useful antigen for detecting and following-up patients with ovarian cancer (Bast *et al.*, 1983; Canney *et al.*, 1984; Schilthuis *et al.*, 1987; Vergote *et al.*, 1987). However, the weak point of this antigen is its relatively low frequency in mucinous-type ovarian cancers and its high false-positive rate especially in patients with endometriosis (Pittaway & Feyez, 1986).

New moAB 130-22 recognising a glycoprotein have been made by Matsuoka *et al.* (1987). CA130 is a glycoprotein recognised by moABs, 130-22 and 145-9, produced by immunisation with human lung adenocarcinoma cell line PC-9. The correlation coefficient between the serum levels of CA130 and CA125 in patients with lung cancer was reported to be 0.8034 (Matsubara *et al.*, 1989). Also, serum CA130 levels in gynaecologic disease were closely correlated with serum CA125 levels, demonstrating quite a high correlation coefficient ($r = 0.965$) (Inaba *et al.*, 1989).

Clinical evaluation of CA130 antigen has been carried out in our hospital. Follow-up studies in some patients showed that CA130 could be useful in monitoring the clinical course of disease after treatment. The value of CA130 in serum as a follow-up marker in ovarian cancer has been pointed out but not yet fully evaluated. This article describes characterisation of the epitopes recognised by the moABs 130-22 and 145-9. The clinical evaluation of CA130 as a tumour marker was investigated by comparison with CA125 in patients with epithelial ovarian cancer and in those with benign gynaecologic disease. The second aim is to evaluate CA130 as a follow-up marker in the treatment of ovarian cancer.

Correspondence: H. Kobayashi, Department of Obstetrics and Gynaecology, Hamamatsu University School of Medicine, Handacho 3600, Hamamatsu, Shizuoka, 431-31, Japan.
Received 8 July 1992; and in revised form 14 September 1992.

### Materials and methods

### *Materials*

Murine moABs OC125 and 130-22/145-9 were donated by Toray Fuji Bionics Co. (Tokyo, Japan) and Daiichi Radioisotope Co. (Tokyo), respectively. All other reagents were of analytical grade.

### *Cell culture*

SHIN-3 cells, established from a patient diagnosed as having serous cystadenocarcinoma of the ovary, were donated by Dr Y. Kiyozuka (Department of Obstetrics and Gynaecology, Kurume University, Japan). SHIN-3 cells produced the maximum amount of CA125 antigen during the growth phase. The production rate of $1 \times 10^6$ cells seeded in a 75-cm$^2$ flask was, reportedly, 75 U ml$^{-1}$ wk$^{-1}$ (Imai *et al.*, 1989; Imai *et al.*, 1990). Another cell line, designated as HOC-I, was established from a recurrent region of ovarian endometrioid carcinoma (Fujii, 1989). The HOC-I cells were grown in RPMI 1640 containing 10% foetal calf serum (FCS). $1 \times 10^6$ cells were seeded in 5 ml medium containing 10% FCS. The production rate of CA125 antigen was approximately 1,700 U ml$^{-1}$ 4 days. To investigate the characteristic of the antigenic determinant of CA130 antigen, the condition media (CM) from two ovarian cancer cell lines were used as the source of CA125 antigen. Pooled culture supernatants were stored at −20°C.

### *Purification of CA125 antigen*

CA125 antigen was purified from the CM of HOC-I ovarian cancer cells according to the method of Davis, H.M. *et al.* (Davis *et al.*, 1986). In brief, pooled CM were filtered and concentrated in an Amicon filter apparatus with a molecular mass cutoff of 30 kDa (Centricon-30). The concentrated CM was subjected to perchloric acid precipitation (0.6 M), then the acid-soluble fraction was neutralised (1N NaOH) and dialysed against water. This sample was applied on a Sephacryl S-300 column, as described previously (Matsuoka *et al.*, 1987). The fraction containing CA125 activity (void volume)

were pooled, dialysed and then concentrated. The sample was treated with urea (6 M, 30 min, 45°C) to disaggregate the high-molecular-mass CA125 molecule, and further fractionation was accomplished by subsequent chromatography on a Sepharose 6B column equilibrated in 50 mM Tris, 6 M urea, 0.1% SDS (pH 8.0). Majority of the CA125 activity was associated with a polydisperse molecular mass of 50 kDa to more than 200 kDa. The fractions containing CA125 activity with molecular mass of equal or greater than 200 kDa (CA125 $\geqslant$200 kDa) and that of less than 200 kDa (CA125 $<$200 kDa) were separately pooled and concentrated. Protein concentration was determined by Lowry's method (1951).

*Reactivity of monoclonal antibodies with cancer cell extracts*

To investigate the characteristic of the CA130 antigen, a competitive inhibition assay was performed. For competition enzyme-linked immunosorbent assay (ELISA), supernatants of sonicated SHIN-3 or HOC-I cells (cancer cell extracts) were coated on a 96 well microtiter plate (Costar, Cambridge, MA) (16 h, 4°C). Increasing concentrations of moABs (0–8.0 $\mu$g ml$^{-1}$) were added to each well (1 h, 23°C). After seven washes, biotin-conjugated second antibody (1.5 $\mu$g ml$^{-1}$, DAKO) was added to each well (1 h, 23°C), followed by incubation with avidin-peroxidase (0.4 $\mu$g ml$^{-1}$, DAKO; 1 h, 23°C). After three washes, wells were incubated with enzyme substrate (3.3′–5.5′ tetramethylbenzidine/ml dimethylsulfoxide) and the absorption was measured in an EIA reader (Model 2550, Bio-Rad, Richmond, California) at 450 nm.

*Reactivity of monoclonal antibodies with trypsin-treated cancer cell extracts*

The microtiter plate was coated with supernatants of sonicated SHIN-3 or HOC-I cells. Each plate was treated with 0.1% trypsin (1 h, 37°C; Sigma). After washing, the reactivity of moABs was investigated as described above.

*Reaction of several sources of CA125 antigen with monoclonal antibodies*

Specificity of each moAB (OC125, 130-22 or 145-9) to HOC-I cell extracts were determined by measuring their ability to inhibit the bindings of moAB by different sources of CA125 antigens. Endometriotic cyst fluid, menstrual blood, amniotic fluid, meconium supernatant (as a negative control), CM from SHIN-3 and HOC-I (crude CA125 antigen), CA125 antigen purified from CM of HOC-I, CA125 $\geqslant$200 kDa, and CA125 $<$200 kDa were used as competitors. To determine if CA125 antigens are involved in the reactivity to moABs, the inhibitory activities of these antigens were assayed. After preincubation of competitors and each moAB (1 h, 23°C), the reaction mixture was transferred to microtiter plates coated with HOC-I cell extracts (1 h, 23°C). Thereafter, the procedure was as described above. 1.3 $\mu$g ml$^{-1}$ of each moAB was used for inhibition assays.

In addition, 96 well microtiter plates coated with 1.0 $\mu$g ml$^{-1}$ immobilised antibody (OC125 or 130-22) were incubated with undiluted serum of patient with ovarian cancer (serum CA125 level = 650 U ml$^{-1}$, 100 $\mu$l, 3 h, 23°C). After five washes, horseradish peroxidase (HRP)-labeled antibody (HRP-OC125 or HRP-145-9) was incubated together with OC125, 130-22, or 145-9 (0–8 $\mu$g ml$^{-1}$), respectively, as a competitor (3 h, 23°C). The enzyme reaction was performed.

*Patient characteristics and serum samples*

Serum samples that had been randomly drawn from 126 patients with epithelial ovarian cancer during the period 1985 through 1991 and from 397 women who were healthy, or possessed benign gynaecologic diseases were obtained from a bank of sera preserved at the Department of Gynaecology, Hamamatsu University School of Medicine, Shizuoka, Japan (Table I). Blood collection was performed within protocols approved by the members of the Gynaecological Cancer Committee of this institute. All patients diagnosed at the Department of Gynaecology, Hamamatsu University hospital and its related hospitals entered the study. In all of the study patients the diagnosis was verified histopathologically. the age of the patients with ovarian cancer at diagnosis ranged from 34 to 73 years (mean age, 58 years). Staging of ovarian cancer according to the FIGO classification showed 45 patients with stage I disease, 20 with stage II, and 61 with stage III. Classified according to histologic type, 69 patients had serous adenocarcinomas, 35 had mucinous adenocarcinoma, ten had clear cell carcinoma, and 12 had endometrioid carcinoma. All patients initially underwent cytoreductive surgery and were treated with five cycles of combination chemotherapy including cisplatin 50 mg m$^{-2}$, adriamycin 50 mg m$^{-2}$, and cyclophosphamide 500 mg m$^{-2}$ (PAC). The study comprises 119 patients whose residual tumour of 2 cm or less in size after primary surgery. Women who did not receive second look operation (SLO) were not eligible for this study. SLO was performed after completion of induction PAC chemotherapy. If macroscopic or microscopic tumour was not detected in specimens, random peritoneal biopsies and peritoneal washings were submitted for cytologic examination. Patients achieving a histologically documented complete response received no further therapy. Patients achieving a surgical complete response (microscopic residual disease) received three additional PAC chemotherapy. Sera were stored at − 80°C until analysis could be performed.

The tumour response was assessed from the computerised tomographic (CT) scan, ultrasonographic (US) examination and routine clinical examinations, with measurement of the product of the largest diameter and the diameter perpendicular to it. Registration of disease progression requires a 25% or more increase in the size of the existing lesions or the appearance of clinically measurable new lesions. Disease regression requires at least a 50% decrease in size for at least 4 weeks for partial response and a disappearance of all clinical signs of malignancy for complete response (Vergote et al., 1987). Patients who died while on study, yet without known progressive disease as defined, were considered to have progression at the date of death.

*Determination of serum CA130 and CA125*

Circulating serum CA130 antigen was assayed in sera by means of a double-determinant sandwich immunoradiometric assay system developed by using two moABs, 130-22 and 145-9 (Matsuoka, 1988). Serum samples were analysed in duplicate. The intra-assay and inter-assay coefficients of variation did not exceed 7.0%. Based on the previous study (Matsuoka et al., 1987; Inaba et al., 1989), an CA130 antigen level $\geqslant$35 U ml$^{-1}$ was defined as elevated.

Serum CA125 assays were performed in duplicate using kits provided by Centocor, Inc (Malvern, PA). A CA125 level of $\geqslant$35 U ml$^{-1}$ was defined as elevated.

The nonparametric Wilcoxon test was used for calculations of statistical significance.

### Results

*Characterisation of antigenic determinant of CA130 antigen*

To investigate the reactivity of each moAB (OC125, 130-22, or 145-9) with ovarian cancer cell extracts, a microtiter plate coated with supernatants of sonicated SHIN-3 or HOC-I cells was incubated with different concentrations (0–8.0 $\mu$g ml$^{-1}$) of moABs as described in Figure 1a,b. OC125, 130-22 and 145-9 reacted with both cancer cell extracts in a dose-dependent manner. The mode of reactivity resembled each other. Trypsin treatment of cancer cell extracts abolished the reactivities of these moABs (Figure 2a,b). To demonstrate the specificity of moAB binding to HOC-I cell extracts, each moAB was preincubated with several CA125 antigens, and then the mixture was added to a microtiter plate. Reactivity

**Figure 1** Reactivities of monoclonal antibodies, OC125, 130-22 and 145-9, with cancer cell extracts. A 96 well microtiter plate coated with supernatants of sonicated SHIN-3 **a**, or HOC-I cells **b**, was incubated with different concentrations of moABs (0–8.0 μg ml$^{-1}$; ●, OC125; ■, 130-22; and □, 145-9).

**Figure 2** Reactivities of monoclonal antibodies, OC125, 130-22 and 145-9, with trypsin-treated cancer cell extracts. A microtiter plate coated with supernatants of sonicated SHIN-3 **a**, or HOC-I cells **b**, was treated with trypsin (0.1%, 1 h, 37°C), and then incubated with different concentrations of moABs (0–8.0 μg ml$^{-1}$; ●, OC125; ■, 130-22; and □, 145-9).

of endometriotic cyst fluid, menstrual blood, amniotic fluid, CM from SHIN-3 and HOC-I, and purified CA125 antigen inhibiting the binding of 130-22 or 145-9 were closely related to that of OC125 (Figure 3). Meconium solution did not react with these moABs. CA125 <200 kDa had a significant inhibitory activity toward the reaction between cancer cell extracts and antibodies (130-22, 145-9 and OC125). The reactivity of 130-22 or 145-9 to cancer cell extracts was slightly decreased by CA125 ⩾200 kDa, whereas the reactivity of OC125 was completely inhibited by CA125 ⩾200 kDa. Comparing the binding of CA125 ⩾200 kDa to OC125 with that to 130-22 or 145-9, CA125 ⩾200 kDa gives rise to more than 1,000-fold decrease in the reactivity with 130-22 or 145-9 as assessed from the concentration giving 50% inhibition of the antigen-antibody reaction in our immunoassay system. The binding of 130-22 or 145-9 was inhibited specifically by CA125 <200 kDa, indicating that an antigenic determinant of CA130 defined by 130-22 and 145-9 were closely related to each other, whereas OC125 recognises a different epitope on the structurally identical molecule. The cross-reactivities of these antibodies were investigated by competitive inhibition assays. In OC125-coated wells, the binding of HRP-labelled OC125 to the CA125 antigen in serum of patient with ovarian cancer was not inhibited with 130-22 and 145-9, and that of HRP-labelled 145-9 to the CA125 antigen was not inhibited with OC125. On the other hand, in 130-22-coated wells, the binding of HRP-labelled OC125 to the CA125 antigen was not inhibited with 130-22 and 145-9, and that of HRP-labelled 145-9 to the CA125 antigen was not inhibited with OC125 (Figure 4). We conclude that the epitope recognised by 130-22 and 145-9 could be separated from that recognised by OC125.

*CA130 as a new tumour marker in ovarian cancer*

We evaluated the significance of tumour marker CA130 in patients with epithelial ovarian cancer and compared the levels with those of the CA125 antigen (Table I). One hundred and fifteen (91.3%) of 126 patients with ovarian cancer were found to have CA130 antigen levels ⩾35 U ml$^{-1}$. The difference in CA130 antigen levels between patients with ovarian cancer and non-malignant subjects was significant ($P < 0.001$). Serum CA125 levels were assayed simultaneously and showed 90.5% positivity in ovarian cancer. In the present study, we noted a higher positiver rate for serum CA125 than previously reported. Most other publications

**Figure 3** Reactivity of several sources of CA125 antigens with moABs determined by inhibition of binding of each moAB to HOC-I cell extracts. Each moAB (1.3 μg ml$^{-1}$) was preincubated with several sources of CA125 antigens (1 h, 23°C) and then the mixture was added to ELISA plate coated with HOC-I cell extract (1 h, 23°C). The following CA125 antigens were used as competitors: endometriotic cyst fluid (○), menstrual blood (●), amniotic fluid (□), meconium solution (■, as a negative control), CM from SHIN-3 (△) and HOC-I (▲), CA125 antigen purified from CM of HOC-I (▽), purified CA125 antigen with molecular mass of less than 200 kDa (CA125 <200 kDa) (▼), purified CA125 antigen with molecular mass of equal or greater than 200 kDa (CA125 ⩾200 kDa) (X). Beginning concentrations of CA125 antigens used in our immunoassay system are that: endometriotic cyst fluid, menstrual blood, amniotic fluid (10% solution in PBS), and meconium solution (1% solution); CM from SHIN-3 and HOC-I cells (undiluted); CA125 antigen purified from CM of HOC-I, CA125 <200 kDa, and CA125 ⩾200 kDa (~0.5 μg ml$^{-1}$).

**Figure 4** The competitive inhibition curves in two-step ELISAs. CA125 antigen was preincubated with each antibody-coated well, and after washing, various concentrations of unlabelled antibodies were added with either HRP-labelled OC125 or HRP-labelled 145-9. **a**, In the OC125/OC125 (capture/tracer) sandwich ELISA, HRP-labelled OC125 was incubated together with OC125 (○), 130-22 (△), or 145-9 (▲) as a competitor. **b**, In the 130-22/145-9 (capture/tracer) sandwich ELISA, HRP-labelled 145-9 was incubated together with OC125 (○), 130-22 (△), or 145-9 (▲) as a competitor. **c**, HRP-labelled 145-9 and OC125-coated wells; and **d**, HRP-labelled 145-9 and 130-22-coated wells.

**Table I** Distribution of serum CA130 and CA125 levels

| | *No. of patients* | *Serum CA130 levels Mean value* ± *s.d. (%)*[a] | *Serum CA125 levels Mean value* ± s.d. (%)[a] |
|---|---|---|---|
| Control | 165 | 12.8 ± 10.6 (9.1) | 18.9 ± 9.8 (9.7) |
| Benign disease | 232 | | |
| Uterine myoma | 63 | 21.6 ± 23.8 (33.3) | 28.3 ± 29.8 (31.7) |
| Endometriosis | 71 | 51.4 ± 48.3 (60.6) | 70.1 ± 69.3 (62.0) |
| Ovarian tumour | 98 | 29.6 ± 30.0 (20.4) | 35.8 ± 31.0 (22.4) |
| Ovarian cancer | 126 | | |
| Stage I | 45 | 119.6 ± 106.3 (84.4) | 169.3 ± 141.2 (82.2) |
| Stage II | 20 | 312.4 ± 281.6 (90.0) | 398.0 ± 350.0 (90.0) |
| Stage III | 61 | 419.3 ± 361.7 (96.7) | 547.4 ± 411.9 (96.7) |

[a]Positive rate (%). The cutoff values for serum CA130 and CA125 have been established as 35 U ml$^{-1}$.

describe an incidence between 80 to 85%. This rather high percentage of patients with elevated serum levels is probably not due to the relatively small number of pre-operative serum samples available for analysis. Some authors reported that pre-operative serum CA125 levels were elevated in 90% or 96% (Kivinen *et al.*, 1986; Shilthuis *et al.*, 1987). We assayed these two antigen levels in sera from patients with benign gynaecologic disease. CA130 and CA125 was positive in 36.2% and 37.1% of patients with benign disease and in 60.6% and 62.0% of those with endometriosis, respectively. Like CA125, CA130 also showed a high false-positive rate in endometriosis in particular. Our data indicated that serum CA130 levels were closely correlated with serum CA125 levels, demonstrating quite a high correlation coefficient ($r = 0.914$). CA130 and CA125 were similar in terms of sensitivity (91.3% *vs* 90.5%) and specificity (75.1% *vs* 74.5%).

Serum CA130 and CA125 levels were assayed simultaneously in 119 patients with residual tumour of 2 cm or less after primary cytoreductive surgery. Results of treatment at SLO are described in Table II. The difference between the CA130 and CA125 levels in pathological complete responders and in surgical complete responders was not significant. In contrast, the differences between these levels in pathological complete responders and in partial responders were significant ($P < 0.05$). Four (10.3%) of 39 patients without residual disease exceeded 35 U ml$^{-1}$ at SLO. For these patients recurrences have not been confirmed for at least 2 years. Six (18.8%) of 32 surgical complete responders, 14 (46.7%) of 30 partial responders, and all of 18 patients with progressive disease showed serum CA130 levels $\geqslant 35$ U ml$^{-1}$.

Thirty-eight (47.5%) of 80 patients with residual disease showed levels of CA130 above the cutoff value. Namely, the sensitivity of CA130 in serum at SLO was 47.5% (38/80) and the specificity was 89.7% (35/39). The positive and negative predictive value of an increase in CA130 at SLO was 90.5% (38/42) and 45.5% (35/77), respectively. CA130 and CA125 yielded essentially identical results in detecting patients with disease at the time of the SLO. We next examined the time when the increase in serum CA130 levels occurs during follow-up (Table III). Sixty-four of 119 patients showed recurrence during follow-up. In 95.3% (61/64) of the patients the CA130 increase was correlated to tumour progression or recurrence.

In 40.6% (26/64) the increase occurred at recurrence, and in 4.7% (3/64) occurred after recurrence and before death. In 50.0% (32/64) there was an increase before recurrence. In three patients (4.7%; two patients with mucinous adenocarcinoma, stage II and one case with clear cell carcinoma, stage III) there were no increase in CA130 levels until death. In one patient with partial response at SLO and in two cases with progressive disease at SLO, a rise in serum CA130 levels predated rising serum CA125 levels. Statistical analysis showed that CA125 and CA130 were similar in terms of the sensitivity, the specificity, the positive and negative predictive value at SLO. Also, the simultaneous assay of CA125 showed that similar results were obtained in terms of the time when the increase in serum CA125 levels occurs during follow-up. A strong correlation was found between CA130 and CA125, suggesting that the use of both assays does not further increase the ability to detect microscopic disease at SLO or during follow-up, since they yielded essentially identical results.

### Discussion

CA130 is a glycoprotein recognised by moABs 130-22 and 145-9. Using these moABs a sensitive sandwich immunoradiometric assay for CA130 was developed (Matsuoka, 1988). CA130 is a new tumour marker, different from CA125, since it has been considered that moABs 130-22 and OC125 recognise distinct antigenic determinants on the same antigen (Endo *et al.*, 1988). The antigenic determinant recog-

**Table II** Serum CA130 and CA125 levels at SLO*

| *Results of treatment at SLO** | *No. of patients* | *Serum CA130 levels at SLO* | | | | *Serum CA125 levels at SLO* | | | |
|---|---|---|---|---|---|---|---|---|---|
| Pathological CR** | 39 | 9.8 ± 6.8*** | (4)**** | | | 14.7 ± 9.1*** | (4)***** | | |
| Surgical CR | 32 | 10.0 ± 8.8 | (6) | a | | 16.2 ± 10.2 | (6) | a | |
| | | | | a | b | | | a | b |
| Partial response | 30 | 24.9 ± 20.1 | (14) | | | 29.5 ± 26.0 | (13) | | |
| | | | | | b | | | | b |
| Progressive disease | 18 | 183.4 ± 265.9 | (18) | | | 223.9 ± 298.4 | (18) | | |

*SLO: Second look operation; **CR: complete response; ***Mean values ± s.d. (U ml); ****No. of patients with CA130 elevation; *****No. of patients with CA125 elevation; a, $P < 0.05$; b, $P < 0.01$. The present study comprises 119 patients whose residual tumour of 2 cm or less in size after primary surgery. All patients initially underwent cytoreductive surgery and were treated with five cycles of combination PAC chemotherapy.

**Table III** Changes in serum CA130 and CA125 levels during follow-up

| Results of treatment at SLO | No. of patients with recurrence during follow-up (%) | The time when the increase in CA130 levels occurs: before recurrence | CA130 elevation: at recurrence | after recurrence | no. CA130 elevation | The time when the increase in CA125 levels occurs: before recurrence | CA 125 elevation: at recurrence | after recurrence | no. CA125 elevation |
|---|---|---|---|---|---|---|---|---|---|
| Pathological CR | 9/39[a] (23.1) | 7 | 2 | 0 | 0 | 7 | 2 | 0 | 0 |
| Surgical CR | 15/32 (46.9) | 5 | 8 | 1 | 1 | 5 | 8 | 1 | 1 |
| Partial response | 22/30 (73.3) | 7[b] | 12 | 1 | 2 | 6 | 13 | 1 | 2 |
| Progressive disease | 18/18 (100) | 13[c] | 4 | 1 | 0 | 11 | 6 | 1 | 0 |
| | 64/119 | 32 (50.0) | 26 (40.6) | 3 (4.7) | 3 (4.7) | 29 (45.3) | 29 (45.3) | 3 (4.7) | 3 (4.7) |

[a]Nine of 39 patients with pathological CR at SLO had recurrence. [b]In one patient, a rise in serum CA130 levels predated rising serum CA125 levels to predict recurrence. [c]In two patients, a rise in serum CA130 levels predated rising serum CA125 levels.

nised by 130-22 or 145-9 contains a protein moiety because the reactivity was greatly diminished by trypsin. To clarify the epitopic structure of CA130, the reactivity of 130-22 or 145-9 with various kinds of CA125 antigens were investigated by a competitive inhibition assay, which revealed that the reactivity of 130-22 or 145-9 was completely inhibited by purified CA125 antigen with molecular mass of less than 200 kDa. Unlike OC125, CA125 antigen with molecular mass of equal or greater than 200 kDa did not strongly inhibit the 130-22 or 145-9 binding. These facts indicated that antigenic determinants of CA125 and CA130 could be separated by treatment of 6 M urea and heating. Also, the result of the cross-reactivities of OC125, 130-22, and 145-9 by competitive inhibition assays indicates that the epitope recognised by 130-22 or 145-9 could be separated from that recognised by OC125. The CA125 molecule is considered to be composed of at least two subunits: one subunit reacts with OC125, and another subunit reacts with 130-22 and 145-9. We do not have evidence at this time, however, that circulating serum CA125 antigen produced by cancer cells is the same as that expressed on their cell surfaces. Nevertheless, moABs 130-22 and 145-9, that are directed at antigen-expressed determinants other than the CA125 determinants, are important for a complete understanding of the cell physiology and the biochemistry of the antigens that expressed the CA125 determinant (Matsuoka *et al.*, 1987).

Coexpression of these new moABs with OC125 in various serum samples, support the notion that the new antibodies recognise epitopes that are closely associated with those recognised by OC125. Serum CA130 levels measured by the assay system were closely correlated with serum CA125 levels (Matsubara *et al.*, 1989; Inaba *et al.*, 1989), demonstrating quite a high correlation coefficient. CA130 thus seems to be a useful new tumour marker for ovarian serous cancer and lung adenocarcinoma (Matsubara *et al.*, 1989; Inaba *et al.*, 1989). The final result is that the antibodies 130-22/145-9 are not superior to OC125 in the detection of epithelial ovarian cancer.

Changes in serum CA125 levels reflect progression or regression of ovarian cancer more than 90% of the time, implying that increases in serum CA125 levels reflect disease progression whereas declines in serum reflect a response to therapy (Canney *et al.*, 1984; Niloff *et al.*, 1986; Kivinen *et al.*, 1986; Vergote *et al.*, 1987; Lavin *et al.*, 1987; Mogensen *et al.*, 1990; Buller *et al.*, 1991). Some authors (Rubin *et al.*, 1989; Buller *et al.*, 1991) and we agree that negative CA125 levels after therapy do not imply no microscopic residual tumour, although for a given individual the change in CA125 level accurately reflects disease status.

In the present study we examined whether our results could give clear indicators as to the clinical value of CA130 determinations in serum during follow-up of patients with ovarian cancer. The positive predictive value was high enough in the individual patients for CA130 to be used as a single predictor of recurrence. A rise at recurrence seems to be more certain than normalisation at response. There was no significant differences between serum CA130 levels in pathological complete responders and those in surgical complete responders at SLO. Like CA125, normalisation of CA130 at response does not imply no microscopic residual disease. It seems that the absence of an increase at recurrence cannot rule out recurrence. Notwithstanding these limitations, CA130 changes during follow-up support the evaluation of recurrence and can be used as a monitoring marker in an individual patient. We conclude that changes in serum CA130 levels during follow-up are closely correlated with those in serum CA125 levels, and that clinical significance of CA130 is almost the same as that of CA125, although these tumour markers have a separate antigenic determinant.

Buller, R.E. *et al.* (1991) assumed that there is a relationship between the amount of residual tumour after cytoreductive surgery and serum CA125 levels, and the decline in serum CA125 levels in patients with effectively treated epithelial ovarian cancer follows an exponential regression. Further prospective clinical trial in a larger series should be carried out in order to investigate the clinical usefulness of CA130 as a follow-up marker in ovarian cancer, and to determine the threshold of the CA130 test distinguishing surgical complete responders from patients with residual disease.

We are greatly indebted to the personnel at Daiichi Radioisotope Co. Ltd. (Tokyo), and Toray Fuji Bionics Co. Ltd (Tokyo), for the extensive assistance provided to us in the measurement of serum CA130 and CA125 antigen levels. We wish to thank Dr K. Sumimoto for the statistical analysis.

### References

BAST Jr, R.C., FEENEY, M., LAZARUS, H., NADLER, L.M., COLVIN, R.B. & KNAPP, R.C. (1981). Reactivity of a monoclonal antibody with human ovarian carcinoma. *J. Clin. Invest.*, **68**, 1331–1337.

BAST, R.C., KLUG, T.L., ST. JOHN, E. & 4 others (1983). A radioimmunoassay using a monoclonal antibody to monitor the course of epithelial ovarian cancer. *N. Engl. J. Med.*, **309**, 883–887.

BULLER, R.E., BERMAN, M.L., BLOSS, J.D., MANETTA, A. & DISAIA, P.J. (1991). CA125 regression: a model for epithelial ovarian cancer response. *Am. J. Obstet. Gynecol.*, **165**, 360–367.

CANNEY, P.A., MOORE, M., WILKINSON, P.M. & JAMES, R.D. (1984). Ovarian cancer antigen CA125: a prospective clinical assessment of its role as a tumor marker. *Br. J. Cancer*, **50**, 765–769.

DAVIS, H.M., ZURAWSKY, V.R., BAST Jr, R.C. & 4 others (1986). Characterization of the CA125 antigen associated with human epithelial ovarian carcinoma. *Cancer Res.*, **46**, 6143–6151.

ENDO, K., MATSUOKA, Y., NAKASHIMA, T. & 5 others (1988). Development of a new sensitive immunoradiometric assay for CA125: mixed use of two monoclonal antibodies reactive with separate epitopes. *J. Tumor Marker Oncol.*, **3**, 65–71.

FUJII, T. (1989). Establishment and characterization of human ovarian endometrioid carcinoma cell line. *Acta. Obstet. Gynaec. Jpn.*, **41**, 161–166.

HAKOMORI, S. (1985). Aberrant glycosylation in cancer cell membranes as focused on glycolipids: overview and perspective. *Cancer Res.*, **45**, 2405–2414.

HERLYN, M., SEARS, H.F., STEPLEWSKI, Z. & 4 others (1982). Monoclonal antibody detection of a circulating tumor-associated antigen. 1. Presence of antigen in sera of patients with colorectal, gastric, and pancreatic carcinoma. *J. Clin. Immunol.*, **2**, 135–140.

IMAI, S., MAEDA, H., KIYOZUKA, Y., NODA, T., MORIYAMA, I. & ICHIJYO, M. (1989). Characterization of the CA125 antigen secreted from a newly established human ovarian cancer cell line (SHIN-3). *Acta Pathol. Jpn.*, **39**, 43–49.

IMAI, S., KIYOZUKA, Y., MAEDA, H., NODA, T. & HOSICK, H.L. (1990). Establishment and characterization of a human ovarian serous cystadenocarcinoma cell line that produces the tumor markers CA125 and Tissue Polypeptide Antigen. *Oncol.*, **47**, 177–184.

INABA, N., OKAJIMA, Y., OTA, Y. & 4 others (1989). A fundamental and clinical investigation of cancer antigen 130 (CA130) in the field of Obstetrics and Gynecology. *J. Jpn. Soc. Cancer. Ther.*, **24**, 2426–2435.

ITZKOWITZ, S.H., YUAN, M. & MONTGOMERY, C.K. (1989). Expression of Tn, sialosyl-Tn, and T antigens in human colon cancer. *Cancer Res.*, **49**, 197–204.

ITZKOWITZ, S.H., BLOOM, E.J. & KOKAL, W.A. (1990). Sialosyl Tn. A novel mucin antigen associated with prognosis in colorectal cancer patients. *Cancer*, **66**, 1960–1966.

KIVINEN, S., KUOPPALA, T., LEPPILAMPI, M., VUORI, J. & KAUPPILA, A. (1986). Tumor-associated antigen CA125 before and during the treatment of ovarian carcinoma. *Obstet. Gynecol.*, **67**, 468–472.

KLUG, T.L., BAST Jr, R.C., NILOFF, J.M., KNAPP, R.C. & ZURAWASKI Jr, V.R. (1984). A monoclonal antibody immunoradiometric assay for an antigenic determinant (CA125) associated with human epithelial ovarian carcinomas. *Cancer Res.*, **44**, 1048–1053.

KOBAYASHI, H., TERAO, T. & KAWASHIMA, Y. (1991). Clinical evaluation of circulating serum sialyl Tn antigen levels in patients with epithelial ovarian cancer. *J. Clin. Oncol.*, **9**, 983–987.

KUZUYA, K., NOZAKI, M. & CHIHARA, T. (1986). Evaluation of CA125 as a circulating tumor marker for ovarian cancer. *Acta. Obstet. Gynaecol. Jpn.*, **38**, 949–957.

LAVIN, P.T., KNAPP, R.C., MALKASIAN, G., WHITNEY, C.W., BEREK, J.C. & BAST Jr, R.C. (1987). CA125 for the monitoring of ovarian carcinoma during primary therapy. *Obstet. Gynecol.*, **69**, 223–227.

LOWRY, O.H., ROSEBROUGH, N.J., FARR, A.L. & RANDALL, R.J. (1951). Protein measurement with the Folin phenol reagent. *J. Biol. Chem.*, **193**, 265–275.

MATSUOKA, Y. (1988). Establishment and clinical application of immunoradiometric assay using two newly developed monoclonal antibodies 130-22 and 145-9. *Mie Igaku*, **32**, 93–99. (in Japanese).

MATSUBARA, Y., SHIOTA, T. & ISHIDA, H. (1989). Clinical evaluation of serum CA130 in patients with lung cancer. *J. Jpn. Soc. Cancer Ther.*, **24**, 1557–1565.

MATSUOKA, Y., NAKASHIMA, T. & ENDO, K. (1987). Recognition of ovarian cancer antigen CA125 by murine monoclonal antibody produced by immunization of lung cancer cells. *Cancer Res.*, **47**, 6335–6340.

MOGENSEN, O., MOGENSEN, B. & JAKOBSEN, A. (1990). Predictive value of CA125 during early chemotherapy of advanced ovarian cancer. *Gynecol. Oncol.*, **37**, 44–46.

NILOFF, J.M., KNAPP, R.C., SCHAERZL, E., REYNOLDS, C. & BAST Jr, R.C. (1984). CA125 antigen levels in obstetric and gynecologic patients. *Obstet. Gynecol.*, **64**, 703–707.

NILOFF, J.M., KNAPP, R.C. & LEVIN, P.T. (1986). The CA125 assay as a predictor of clinical recurrence in epithelial ovarian cancer. *Am. J. Obstet. Gynecol.*, **155**, 56–60.

PITTAWAY, D.E. & FAYEZ, J.A. (1986). The use of CA125 in the diagnosis and management of endometriosis. *Fertil. Steril.*, **46**, 790–795.

RUBIN, S.C., HOSKINS, W.J. & HAKES, T.B. (1989). Serum CA125 levels and surgical findings in patients undergoing secondary operations for epithelial ovarian cancer. *Am. J. Obstet. Gynecol.*, **160**, 667–671.

SCHILTHUIS, M.S., AALDERS, J.G. & BOUMA, J. (1987). Serum CA125 levels in epithelial ovarian cancer: relation with findings at second-look operations and their role in the detection of tumor recurrence. *Br. J. Obstet. Gynecol.*, **94**, 202–207.

SPRINGER, G.F. (1984). T and Tn, General carcinoma autoantigens. *Science*, **224**, 1198–1206.

SPRINGER, G.F., DESAI, P.R. & ROBINSON, M.K. (1986). The fundamental and diagnostic role of T and Tn antigens in breast carcinoma at earliest histologic stage and throughout. In Dao, T., Brodie, A. and Ip, C. (eds), *Tumour Markers and Their Significance in the Management of Breast Cancer*, New York: A.R. Liss., pp. 47–70.

VERGOTE, I.B., BORMER, O.P. & ABELER, V.M. (1987). Evaluation of serum CA125 levels in the monitoring of ovarian cancer. *Am. J. Obstet. Gynecol.*, **157**, 88–92.

ZANABONI, F., VERGADORO, F., PRESTI, M., GALLOTTI, P., LOMBARDI, F. & BOLIS, G. (1987). Tumor antigen CA125 as a marker of ovarian epithelial carcinoma. *Gynecol. Oncol.*, **28**, 61–67.